# Cytological studies of *Rungia* (Acanthaceae) from China and its systematic implications

**DOI:** 10.3897/phytokeys.277.199752

**Published:** 2026-07-16

**Authors:** Zhe-Li Lin, Shu Wang, Yun-Fei Deng

**Affiliations:** 1 Guangdong Provincial Key Laboratory of Utilization and Conservation of Food and Medicinal Resources in Northern Region/College of Biology and Agriculture, Shaoguan University, Shaoguan 512005, China State Key Laboratory of Plant Diversity and Specialty Crops, South China Botanical Garden, Chinese Academy of Sciences Guangzhou China; 2 State Key Laboratory of Plant Diversity and Specialty Crops, South China Botanical Garden, Chinese Academy of Sciences, Guangzhou, Guangdong 510650, China Guangdong Provincial Key Laboratory of Utilization and Conservation of Food and Medicinal Resources in Northern Region/College of Biology and Agriculture, Shaoguan University Shaoguan China; 3 Key Laboratory of National Forestry and Grassland Administration on Plant Conservation and Utilization in Southern China, Guangzhou 510650, China Key Laboratory of National Forestry and Grassland Administration on Plant Conservation and Utilization in Southern China Guangzhou China

**Keywords:** Basic chromosome number, infrageneric classification, *
Rungia
*, taxonomy

## Abstract

In this study, the somatic chromosome numbers for 30 populations representing 14 *Rungia* species from China were investigated. Chromosome counts for eleven of these species are reported for the first time, excluding *Rungia pectinata*, *R. hirpex* and *R. flaviflora*. The genus presents rich cytological diversity, with chromosome numbers of 2*n* = 20, 22, 26, 28, 40, 42, 50, 52 and 56. Polyploidy is prevalent, occurring in ten populations representing six species and accounting for approximately 33% of the studied populations. Based on the integration of the present data with previous reports, five basic chromosome numbers (*x* = 10, 11, 13, 14 and 25) were identified for the 16 species with available data. This pattern of base numbers is largely congruent with some key morphological data, such as capsule type and anther size. These findings confirm the significance of chromosomal data in the systematic analysis of *Rungia*, particularly for infrageneric classification, while also highlighting the need for further molecular and micromorphological studies to draw more comprehensive conclusions.

## Introduction

*Rungia* Nees (Acanthaceae, Justicieae) comprises approximately 50 species distributed through tropical and subtropical regions of the Old World (Mabberley 2017). In China, sixteen species of *Rungia* were recognised in the *Flora of China* (Hu et al. 2011). Recently, the species of *Rungia* distributed in China have been revised. *Rungia monetaria* (Benoist) B. Hansen is a misidentification of *R. flaviflora* Z.L. Lin & Y.F. Deng and should be excluded from China (Lin and Deng 2018); in addition, *R. axilliflora* and *R. densiflora* have been treated as synonyms of *R. stolonifera* C.B. Clarke (Lin et al. 2020). Meanwhile, *R. burmanica* (C.B. Clarke) B. Hansen was reported as a new record in China (Lin and Deng 2017a, 2018) and three new species, viz. *R. sinothailandica* Z.L. Lin & Y.F. Deng, *R. flaviflora* Z.L. Lin & Y.F. Deng and *R. fangdingiana* Z.L. Lin, Y.F. Deng & Y.H. Tan, were recently described (Lin and Deng 2017b, 2018; Lin et al. 2022). Furthermore, two additional species, *R. khasiana* T. Anderson and *R. apiculata* Bedd., have been discovered as new records in China (Lin & Deng, to be published). Consequently, the genus *Rungia* is currently represented by a total of nineteen species in China.

*Rungia* has traditionally been considered most closely related to *Justicia* L., from which it is primarily distinguished by the presence of elastic placentae (Hansen 1989; Hu 2002; Hu et al. 2011; Deng and Gao 2020). Some authors (Darbyshire et al. 2010; Vollesen 2015; Wood 2014) considered that an elastic placenta is not a diagnostically stable character for distinguishing the two genera and, therefore, merged *Rungia* with *Justicia*, with transferring some species to *Justicia*. However, the recent phylogenetic studies (Deng et al. 2016; Kiel et al. 2017; Niu et al. 2023) have shown that *Justiciasensu lato* is not monophyletic and the genus *Rungia* should be re-instated as a distinct genus.

Based mainly on inflorescence symmetry and bract fertility, Gao and Deng (2007) divided the genus *Rungia* into two sections, i.e. *R.* sect. *Stoloniferae* C.M. Gao & Y.F. Deng, characterised by symmetrical spikes with four-ranked, entirely fertile bracts, comprising two species; and *R.* sect. *Rungia*, defined by secund spikes with four-ranked bracts, two fertile and two sterile, including the remaining species. However, a more comprehensive evidence base is required to evaluate and refine the infrageneric classification.

Chromosomal data are of great significance in the study of plant systematics and species delimitation (Hong 1990; Watanabe et al. 1995; Liu and Yang 2011). Despite their potential systematic importance in *Rungia*, cytological studies in this genus remain limited. To date, chromosome numbers have been documented for only five species (Table 1) and, aside from these five species (representing less than 10% of the genus), the remaining members of *Rungia* have not yet been cytologically investigated.

**Table 1.** An integrative analysis of chromosomal data in the genus *Rungia*.

**Taxon**	**Locality**	**Voucher in present study (or references in previous reports)**	**Basic chromosome number**	**2*n***	**Ploidy level**	**Figure**	**Infrageneric classification of *Rungia* (Gao and Deng 2007)**
*R. repens	India	Narayanan 1951; Ellis 1962; Bhat and Tandon 1967; Ranganath 1981; Krishnappa and Basavaraj 1982; Saggoo 1983	10	20	2x		Sect. *Rungia*
	India	(Subramanian and Govindarajan 1980; Govindarajan and Subramanian 1983)		(2*n* = 34, 36)			
***R. khasiana***	Mengla, Yunnan	*Z. L. Lin & X. E. Ye 14032101*	10	20	2x	1A	Sect. *Rungia*
***R. apiculata***	Baisha, Hainan	*Z. L. Lin & Q. L. Wang 14022301*	10	40	4x	1B	Sect. *Rungia*
***R. bisaccata***	Pingxiang, Guangxi	*Z. L. Lin & X. Q. Guo L15050501*	11	22	2x	1C	Sect. *Rungia*
***R. burmanica***	Maguan, Yunnan	*Z. L. Lin & F. Peng 14121910*	11	22	2x	1D	Sect. *Rungia*
*R. flaviflora	Gejiu, Yunnan	*Z. L. Lin L15032802*; Lin and Deng 2018	11	22	2x	1E	Sect. *Rungia*
***R. pinpienensis***	Maguan (Gulinqing), Yunnan	*Z. L. Lin & F. Peng 14121918*	11	22	2x	1F	Sect. *Rungia*
	Maguan (Miechang), Yunnan	*Z. L. Lin & F. Peng 14122012*	11	22	2x	1G	Sect. *Rungia*
***R. sinothailandica***	Jinghong, Yunnan	*Z. L. Lin & Y. Y. Shao L16032701*	11	22	2x	1H	Sect. *Rungia*
*R. laeta	India	Saggoo and Bir 1982a, 1986; Saggoo 1983	13	26	2x		Sect. *Rungia*
***R. stolonifera***	Puer, Yunnan	*Z. H. Li LZH007*	13	26 + 2Bs	2x	1L	Sect. *Stoloniferae*
*R. axilliflora* (synonym of *R. stolonifera*)	Leye, Guangxi	*Z. L. Lin & X. Q. Tian L15100501*	13	26 + 2Bs	2x	1I	Sect. *Stoloniferae*
	Xingyi, Guizhou	*Z. L. Lin & F. Peng 14102301*	13	26 + 2Bs	2x	1J	Sect. *Stoloniferae*
*R. densiflora* (synonym of *R. stolonifera*)	Lianping, Guangdong	*Z. L. Lin L15101801*	13	26 + 2Bs	2x	1K	Sect. *Stoloniferae*
*R. pectinata	Baisha, Hainan	*Z. L. Lin & Q. L. Wang 14022302*	13	26	2x	2A	Sect. *Rungia*
	Jinghong, Yunnan	*Z. L. Lin & Y. Y. Shao L16032501*	13	26	2x	2B	Sect. *Rungia*
	Mengla, Yunnan	*Z. L. Lin & X. E. Ye 14031902*	13	52	4x	2C	Sect. *Rungia*
	Puer, Yunnan	*Z. L. Lin & Y. Y. Shao L16032902*	13	52	4x	2D	Sect. *Rungia*
	Asia	Bir and Saggoo 1979, 1981; Sarkar et al. 1980; Saggoo and Bir 1982b; Saggoo 1983, 1987	13	26	2x		Sect. *Rungia*
	Asia	Mehra and Vasudevan 1972; Sareen and Sanjota 1976; Vasudevan 1976; Bir and Saggoo 1979; Sarkar et al. 1980; Bir et al. 1982; Saggoo 1983, 1987	13	52	4x		Sect. *Rungia*
	Asia	(De 1966; Baquar 1967–1968; Datta and Maiti 1970; Devi 1980)		(*n* = 8, 15; 2*n* = 16, 30, 50, 60)			
***R. chinensis***	Gejiu, Yunnan	*Z. L. Lin & F. Peng 14122203*	13	52	4x	2E	Sect. *Rungia*
	Huanjiang, Guangxi	*Z. L. Lin & X. Q. Tian L15100301*	13	52	4x	2F	Sect. *Rungia*
	Xingyi, Guizhou	*Z. L. Lin & F. Peng 14102313*	13	52	4x	2G	Sect. *Rungia*
	Zhaoqing, Guangdong	*Z. L. Lin 14091801*	13	52	4x	2H	Sect. *Rungia*
*R. hirpex	Yanjin, Yunnan	*Z. L. Lin & X. Q. Tian L15092401*; Lin et al. 2016	13	52 + 1SAT	4x	2I	Sect. *Rungia*
***R. pungens***	Napo (Baidu), Guangxi	*Z. L. Lin & X. Q. Guo L15050701*	14	28	2x	3A	Sect. *Rungia*
	Napo (Bainan), Guangxi	*Z. L. Lin & X. D. Ma 13123004*	14	28	2x	3B	Sect. *Rungia*
	Longzhou, Guangxi	*Z. L. Lin & Z. X. Zhang 14030601*	14	42	3x	3C	Sect. *Rungia*
***R. yunnanensis***	Gejiu, Yunnan	*Z. L. Lin & F. Peng 14122201*	14	28	2x	3D	Sect. *Rungia*
	Maguan (Bojia), Yunnan	*Z. L. Lin & X. D. Ma 14010602*	14	28	2x	3E	Sect. *Rungia*
	Maguan (Tuanjie), Yunnan	*Z. L. Lin & F. Peng 14121923*	14	28	2x	3F	Sect. *Rungia*
	Mengla, Yunnan	*Z. L. Lin & X. E. Ye 14031901*	14	28	2x	3G	Sect. *Rungia*
	Jiangcheng, Yunnan	*Z. L. Lin & X. E. Ye 14032522*	14	56	4x	3H	Sect. *Rungia*
***R. mina***	Puer, Yunnan	*Z. L. Lin & F. Peng 14101801*	25	50	2x	3I	Sect. *Stoloniferae*

* Previous reports. Counts in parentheses are likely erroneous reports.

In this study, we conducted an extensive survey of chromosome numbers in Chinese *Rungia*, to determine the basic chromosome numbers and to evaluate the significance of chromosomal data for systematic study in this genus.

## Materials and methods

The materials studied were collected from the field and cultivated in the greenhouse of South China Botanical Garden, the Chinese Academy of Sciences and Shaoguan University. The voucher specimens (Table 1) are deposited in the Herbarium of South China Botanical Garden, Chinese Academy of Sciences (IBSC).

For the chromosome studies, root tips were pretreated in a 1:1 mixture of 0.1% colchicine and 0.002 M 8-hydroxyquinoline for about 2.5 hours, then fixed in Carnoy’s fluid (glacial acetic acid:absolute ethanol = 1:3) for at least 2 hours. The fixed roots were macerated in 1 M hydrochloric acid (HCl) at 37 °C for 45 minutes, then stained in Carbol fuchsin and squashed for observation in a microscope. We followed Liu and Yang (2011) with minor modifications. The mitotic metaphase chromosomes of at least 30 cells (from 3–5 individuals) were counted and photographed.

## Results

**Figure 1.**
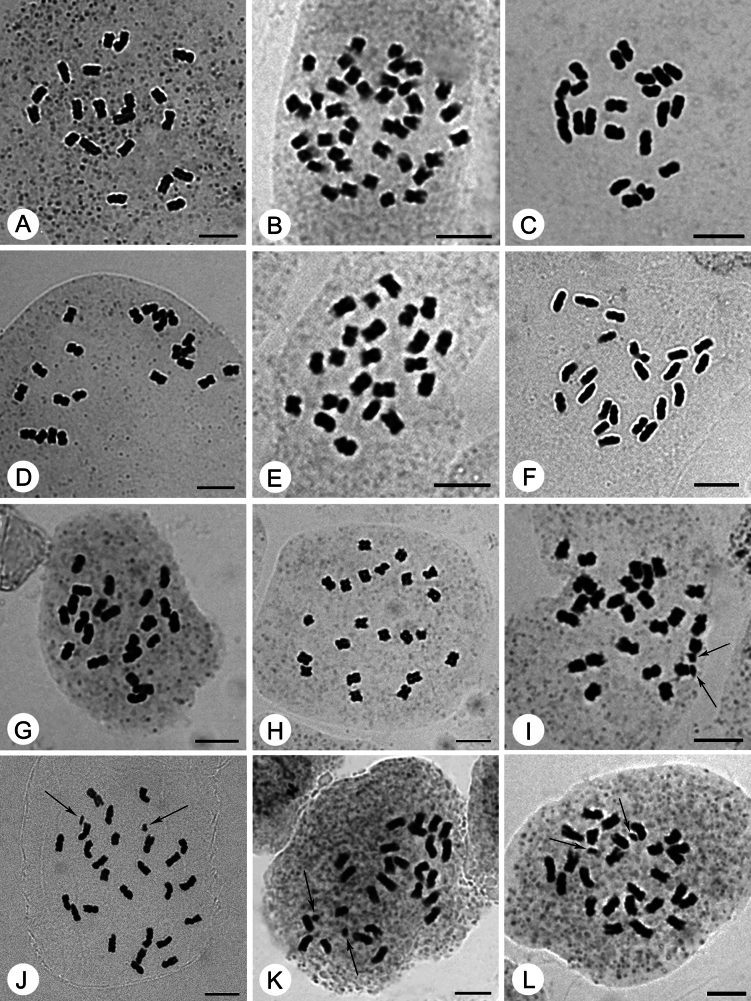
Mitotic metaphase chromosomes in *Rungia*. **A**. *R. khasiana* (Mengla), 2*n* = 20; **B**. *R. apiculata* (Baisha), 2*n* = 40; **C**. *R. bisaccata* (Pingxiang), 2*n* = 22; **D**. *R. burmanica* (Maguan), 2*n* = 22; **E**. *R. flaviflora* (Gejiu), 2*n* = 22; **F**. *R. pinpienensis* (Gulinqing, Maguan), 2*n* = 22; **G**. *R. pinpienensis* (Miechang, Maguan), 2*n* = 22; **H**. *R. sinothailandica* (Jinghong), 2*n* = 22; **I**. *R. stolonifera* (Leye: the type locality of *R. axilliflora*), 2*n* = 26 + 2Bs; **J**. *R. stolonifera* (Xingyi: near the type locality of *R. axilliflora*), 2*n* = 26 + 2Bs; **K**. *R. stolonifera* (Lianping: the type locality of *R. densiflora*), 2*n* = 26 + 2Bs; **L**. *R. stolonifera* (Puer), 2*n* = 26 + 2Bs. Arrows indicate B chromosomes. Scale bars: 5 μm.

**Figure 2.**
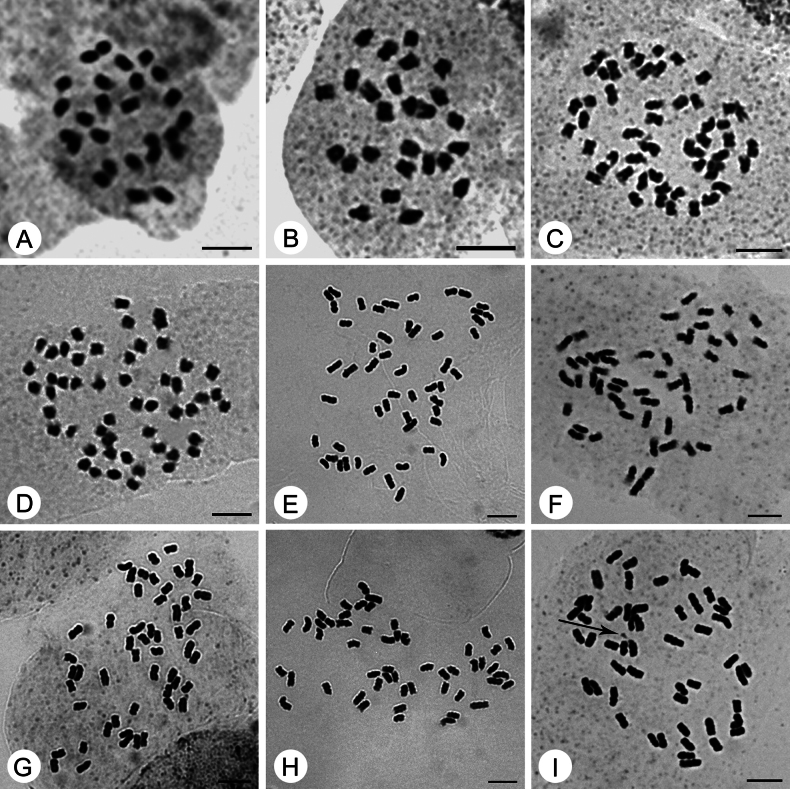
Mitotic metaphase chromosomes in *Rungia*. **A**. *R. pectinata* (Baisha), 2*n* = 26; **B**. *R. pectinata* (Jinghong), 2*n* = 26; **C**. *R. pectinata* (Mengla), 2*n* = 52; **D**. *R. pectinata* (Puer), 2*n* = 52; **E**. *R. chinensis* (Gejiu), 2*n* = 52; **F**. *R. chinensis* (Huanjiang), 2*n* = 52; **G**. *R. chinensis* (Xingyi), 2*n* = 52; **H**. *R. chinensis* (Zhaoqing), 2*n* = 52; **I**. *R. hirpex* (Yanjin), 2*n* = 52 + 1SAT. Arrows indicate satellite. Scale bars: 5 μm.

**Figure 3.**
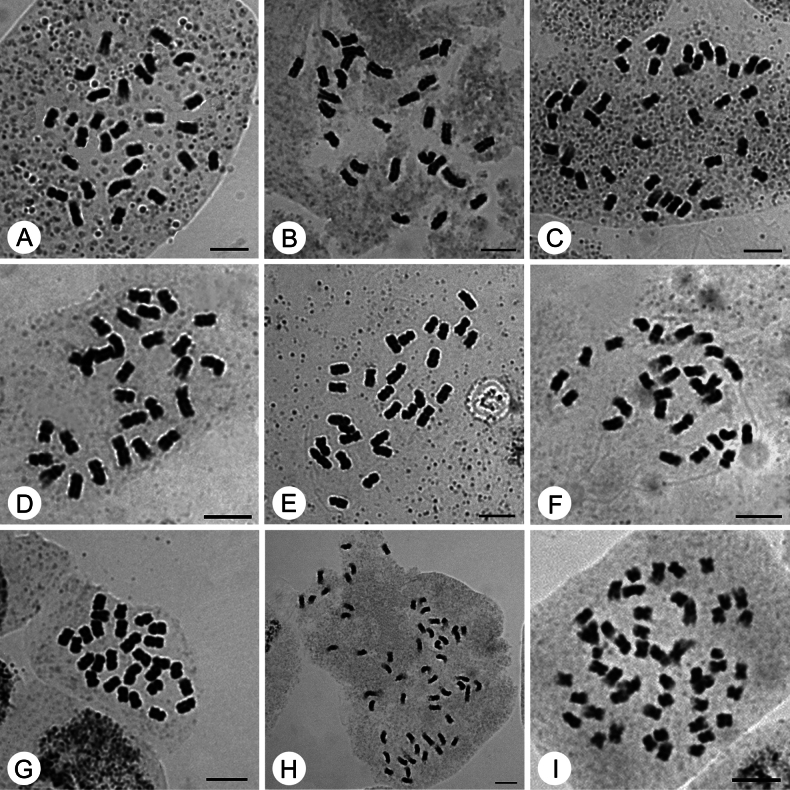
Mitotic metaphase chromosomes in *Rungia*. **A**. *R. pungens* (Baidu, Napo), 2*n* = 28; **B**. *R. pungens* (Bainan, Napo), 2*n* = 28; **C**. *R. pungens* (Longzhou), 2*n* = 42; **D**. *R. yunnanensis* (Gejiu), 2*n* = 28; **E**. *R. yunnanensis* (Bojia, Maguan), 2*n* = 28; **F**. *R. yunnanensis* (Tuanjie, Maguan), 2*n* = 28; **G**. *R. yunnanensis* (Mengla), 2*n* = 28; **H**. *R. yunnanensis* (Jiangcheng), 2*n* = 56; **I**. *R. mina* (Puer), 2*n* = 50. Scale bars: 5 μm.

This paper presents the first comprehensive survey of chromosome numbers in Chinese *Rungia*. The chromosome numbers of 30 populations representing 14 species of *Rungia* from China were studied (Table 1), with chromosomal morphology and size illustrated in Figs 1, 2, 3. Amongst these, chromosome numbers are reported for the first time for eleven species. The results are summarised as follows:

*Rungia khasiana* T. Anderson had a chromosome count of 2*n* = 20 in one population from Yunnan Province.

*Rungia apiculata* Bedd. had 2*n* = 40 in one population from Hainan Province.

Five species had the chromosome number of 2*n* = 22, i.e. *Rungia bisaccata* D. Fang & H.S. Lo in one population from Guangxi, *R. burmanica* (C.B. Clarke) B. Hansen in one population from Yunnan, *R. flaviflora* Z.L. Lin & Y.F. Deng in one population from Yunnan, *R. pinpienensis* H.S. Lo in two populations from Yunnan and *R. sinothailandica* Z.L. Lin & Y.F. Deng in one population from Yunnan.

One species, *Rungia pectinata* (L.) Nees, had both 2*n* = 26 and 52. The chromosome number of 2*n* = 52 was counted in two populations, one from Yunnan and the other from Hainan, and 2*n* = 26 was counted in two populations from Yunnan.

*Rungia stolonifera* C.B. Clarke was found to have 2*n* = 26 + 2B, based on studies of four populations from Guangdong, Guangxi, Guizhou and Yunnan. Notably, the population from Guangxi was collected from the type locality of *R. axilliflora*, while that from Guangdong was collected from the type locality of *R. densiflora*, these two species being synonyms of *R. stolonifera*.

*Rungia chinensis* Benth. had 2*n* = 52 in four populations each from Guangdong, Guangxi, Guizhou and Yunnan.

*Rungia hirpex* Benoist exhibited 2*n* = 52 + 1SAT in one population from Yunnan.

*Rungia pungens* D. Fang & H.S. Lo had both 2*n* = 28 and 42, the chromosome number of 2*n* = 28 was observed in two populations from Guangxi and 2*n* = 42 was observed in one populations from Guangxi.

*Rungia yunnanensis* H. S. Lo had both 2*n* = 28 and 56, four populations with 2*n* = 28 and one with 2*n* = 56, all from Yunnan.

*Rungia mina* H. S. Lo had 2*n* = 50 in one population from Yunnan.

B chromosomes were observed in *Rungia stolonifera* (Fig. 1I–L). These B chromosomes were markedly smaller than the autosomes and were numerically stable across the four populations studied, as well as within populations.

A satellite was detected in *Rungia hirpex* (Fig. 2I). This represents the only observation of a satellite amongst the *Rungia* species examined.

## Discussion

### Basic chromosome numbers in *Rungia*

Chromosomal data have previously been documented for only five species of *Rungia*. Amongst these, *R. laeta* has multiple consistent counts of *n* = 13, single counts have been reported for *R. hirpex* as 2*n* = 52 and *R. flaviflora* as 2*n* = 22, whereas *R. repens* and *R. pectinata* have multiple, though often conflicting reports. For *R. repens*, the reports include *n* = 10; 2*n* = 20, 34 and 36, while for *R. pectinata*, an even wider array has been recorded: *n* = 8, 13, 15 and 26; 2*n* = 16, 26, 30, 50, 52 and 60 (see references in Table 1).

The chromosome counts for *Rungia laeta* is reliable due to multiple consistent reports, and we are able to confirm the counts for *R. hirpex* and *R. flaviflora* with clear metaphase chromosome photographs. As for *R. pectinata*, we examined four populations and consistently observed only 2*n* = 26 and 52. This finding casts serious doubt on the earlier reports of *n* = 8, 15; 2*n* = 16, 30, 50 and 60 for *R. pectinata*, which we suspect are attributable to misidentification or counting errors. As for *R. repens*, we examined its close relatives, *R. khasiana* and *R. apiculata*, which are shown to have 2*n* = 20 and 40, respectively. Importantly, neither 2*n* = 34 nor 36 has ever been recorded elsewhere in *Rungia*, either in previous literature or in our study. The genuine chromosome number for *R. repens* is thus most likely 2*n* = 20.

In summary, chromosomal data are now available for 16 species in the genus, including the eleven species reported here for the first time. The reliable chromosome numbers for *Rungia* are 2*n* = 20, 22, 26, 28, 40, 42, 50, 52 and 56, yielding an estimated set of base chromosome numbers as *x* = 10, 11, 13, 14 and 25. Amongst these, *x* = 11 and 13 are the most common, each occurring in five species; *x* = 10 appears in three species, *x* = 14 in two and *x* = 25 is found only in one species. Such a wide range of variation in base numbers strongly suggests that *Rungia* is cytologically highly evolved, likely as a result of aneuploidy, a process that may have driven the evolution of morphological variation (Saggoo and Bir 1982b).

### Polyploidy in *Rungia*

The results demonstrate that polyploidy is prevalent in *Rungia*, occurring at both interspecific and intraspecific levels. Amongst species with *x* = 10, *R. apiculata* exhibits a tetraploid cytotype (2*n* = 4*x* = 40). Within the *x* = 13 group, *R. pectinata* comprises both diploid (2*n* = 2*x* = 26) and tetraploid (2*n* = 4*x* = 52) cytotypes, while *R. chinensis* (stable across four populations) and *R. hirpex* are consistently tetraploid (2*n* = 4*x* = 52). Amongst species with *x* = 14, *R. pungens* includes both diploid (2*n* = 2*x* = 28) and triploid (2*n* = 3*x* = 42) cytotypes, and *R. yunnanensis* contains both diploid (2*n* = 2*x* = 28) and tetraploid (2*n* = 4*x* = 56) cytotypes. Notably, all species with *x* = 11 maintain a diploid cytotype (2*n* = 2*x* = 22).

In total, polyploidy was observed in ten populations representing six species, accounting for approximately 33% of the total populations studied. It can be anticipated that further investigations will reveal additional polyploid populations or species within the genus. These findings suggest that polyploidy has likely played an important role in the speciation of *Rungia*.

### B chromosomes in *Rungia*

In the present study, B chromosomes were observed exclusively in four populations of *Rungia stolonifera* (2*n* = 2*x* = 26 + 2Bs). Notably, the number of B chromosomes was consistently two across all populations examined. This cytological stability further supports the recent synonymisation of *R. axilliflora* and *R. densiflora* under *R. stolonifera* (Lin et al. 2020). We also predict that *R. evrardii* Benoist (a species from Vietnam recently merged into *R. stolonifera*) may likewise possess a chromosome number of 2*n* = 26 + 2Bs.

Previous studies have reported 0–3 B chromosomes in *R. pectinata* (Bir and Saggoo 1979; Bir et al. 1982; Saggoo 1983, 1987). However, these remain uncertain due to the absence of clear cytological documentation in some reports, warranting further verification.

### Satellite in *Rungia*

Based on present and previous studies, *Rungia hirpex* is the only species in the genus reported to possess a satellite (Lin et al. 2016). Although *R. chinensis* (2*n* = 52) and *R. hirpex* (2*n* = 52 + 1SAT) share similar chromosome numbers and macromorphology, they can be distinguished by bract and floral characteristics. The presence of a satellite in *R. hirpex* provides additional cytological evidence supporting their distinction as separate species.

### Systematic implications of chromosomal data in *Rungia*

To evaluate the validity of the current infrageneric classification of *Rungia* against chromosomal data, we compared the classification established by Gao and Deng (2007) with the distribution of basic chromosome numbers (Table 1). The distinction between Sect. *Rungia* and sect. *Stoloniferae*, which is based primarily on inflorescence symmetry and bract fertility, is not reflected in the pattern of basic chromosome numbers. As shown in Table 1, Sect. *Rungia* (defined by spike secund; bracts 4-ranked, with two fertile ranks and two sterile ranks) comprises species with four distinct basic chromosome numbers: (1) the *x* = 10 group, including *R. repens*, *R. khasiana* and *R. apiculata*; (2) the *x* = 11 group, including *R. bisaccata*, *R. burmanica*, *R. flaviflora*, *R. pinpienensis* and *R. sinothailandica*; (3) the *x* = 13 group, including *R. laeta*, *R. pectinata*, *R. chinensis* and *R. hirpex*; (4) the *x* = 14 group, including *R. pungens* and *R. yunnanensis*. Sect. *Stoloniferae* (defined by spike symmetrical; bracts 4-ranked, all fertile) contains only two species, *R. stolonifera* with *x* = 13 and *R. mina* with *x* = 25.

Notably, the grouping of *Rungia* species into these five cytological assemblages (*x* = 10, 11, 13, 14 and 25) is strongly supported by several macromorphological and micromorphological traits, such as capsule type, anther size and seed testa structure. Furthermore, the species with *x* = 25 is morphologically very similar to those with *x* = 13 in terms of both macromorphological and micromorphological characters and these two groups are considered to be most closely related to each other based on morphology (Lin & Deng, in preparation). Based on morphological affinities correlated with chromosome numbers, we predict the following base numbers for the five Chinese species not yet cytologically examined: *R. taiwanensis* likely has *x* = 13, while *R. fangdingiana*, *R. napoensis*, *R. longipes* and *R. guangxiensis* are predicted to have *x* = 11.

Several lines of evidence suggest that the current infrageneric classification may be unnatural. For instance, all species with *x* = 13 (and 25) possess an ovoid, unstipitate capsule, whereas those with *x* = 10, 11 and 14 consistently have clavate, stipitate capsules. However, under the current system, the five species with *x* = 13 analysed here are split between two sections, based on inflorescence and bract characters (a treatment that conflicts with both chromosomal and capsule morphological data). Similarly, all species with *x* = 11 possess larger anthers (ca. 3 mm long), while those with other base numbers have smaller anthers (< 2 mm long). Yet, the current classification does not segregate the *x* = 11 group from the other cytological assemblages.

In conclusion, from a cytological perspective, the species of *Rungia* can be divided into five assemblages, the *x* = 10, *x* = 11, *x* = 13, *x* = 14 and *x* = 25 groups. This division is supported by key morphological characters of the anther, capsule and seed. Our findings indicate that chromosomal data are significant for the systematics of *Rungia* and play an important role in evaluating the current infrageneric classification. However, a limitation of this study is the restricted geographic sampling, which included only Chinese species and two from India. Future studies incorporating chromosome counts from other regions would provide a more comprehensive perspective.

Given the incongruence between the current infrageneric system and the cytological and morphological evidence, we recommend that future classifications integrate more comprehensive data, including molecular and micromorphological evidence, to establish a more natural system for the genus.

## Additional information

### Artificial Intelligence (AI) use

The authors accept full responsibility for the content of the manuscript, including the disclosure of any use of AI.

No AI tools were used in the preparation of this manuscript.

### Author ORCIDs

Z.-L. Lin https://orcid.org/0000-0003-2832-8115

### Data availability

All of the data that support the findings of this study are available in the main text.
